# CTRIAL-IE (ICORG) 15–34: The impact of the 21 gene breast recurrence score® assay on chemotherapy prescribing in oestrogen receptor positive, lymph node positive early stage breast cancer in Ireland

**DOI:** 10.1007/s11845-025-03922-7

**Published:** 2025-04-11

**Authors:** W. J. Mullally, A. Hassan, N. Keegan, C. O’Leary, L. McSorley, T. Mahgoub, S. O’Reilly, J. Walshe, M. J. Kennedy, L. Coate, M. O’Connor, M. Keane, C. M. Kelly, K. Duffy, C. G. Murphy, M. Milewski, S. Molloy, K. Egan, V. Murphy, O. S. Breathnach, L. Grogan, B. T. Hennessy, P.G. Morris

**Affiliations:** 1 Cancer Clinical Trials and Research Unit, Beaumont RCSI Cancer Centre, Dublin 9, D09 V2N0 Dublin, Ireland; 2 Department of Medical Oncology, Cork University Hospital, T12 DC4A Cork, Ireland; 3 Department of Medical Oncology, St Vincent’S University Hospital, D04 T6F4 Dublin, Ireland; 4 Department of Medical Oncology, St James’s Hospital, Dublin 8, D08 NHY1 Dublin, Ireland; 5 Department of Medical Oncology, University Hospital Limerick, V94 F858 Limerick, Ireland; 6 Department of Medical Oncology, University Hospital Waterford, X91 ER8E Waterford, Ireland; 7 Department of Medical Oncology, University Hospital Galway, H91 YR71 Galway, Ireland; 8 Department of Medical Oncology, Mater Misericordiae University Hospital University, Dublin 7, D07 AX57 Dublin, Ireland; 9 Department of Medical Oncology, Letterkenny University Hospital, F92 AE81 Letterkenny, Co. Donegal Ireland; 10 Department of Medical Oncology, Bon Secours Hospital, T12 DV56 Cork, Cork Ireland; 11 Cancer Trials Ireland, Dublin, Ireland

**Keywords:** Adjuvant chemotherapy, Breast cancer, Endocrine therapy, Oncotype DX, Recurrence score

## Abstract

**Background:**

The 21-gene Breast Recurrence Score® (Oncotype DX®) assay has improved the selection of patients for chemotherapy in early breast cancer. Internationally, this test is used in lymph node positive disease, but at the time this study was conducted, it was not reimbursed for this indication in Ireland.

**Aims:**

Determine how access to the Recurrence Score® testing reduces chemotherapy use and quantifies the impact on oncologists’ treatment recommendations.

**Methods:**

Between March and September 2017, 75 patients were enrolled in a prospective study across ten hospitals. Eligible patients had oestrogen/progesterone receptor positive and HER2 negative breast cancer with 1–3 involved lymph nodes. Following informed consent, demographics were collected and questionnaires completed by a consultant medical oncologist before and after the recurrence score testing, which examined expectations of tumour chemosensitivity, strength of chemotherapy recommendation, and planned treatment.

**Results:**

Recurrence Scores® were available on 74/75 patients. Overall, access to this test led to a 27% reduction in the recommendation for chemotherapy from 68 (92%) to 48 (65%) patients. This was most notable in patients with one (46 versus 34 patients) and two (13 versus seven patients) involved lymph nodes representing a 26% and 46% reduction, respectively. The reduction in chemotherapy use was marked in women aged 50–70 years with one lymph node involved (28 versus 18 patients)—a 36% reduction.

**Conclusion:**

Consistent with our hypothesis, broader access to the Recurrence Score® led to a reduction in the use of chemotherapy in Ireland and has subsequently become standard of care.

## Background

Breast cancer is the second most common cancer in the world and the fifth cause of mortality worldwide [1]. Primary breast cancer is a highly heterogeneous disease, and increasingly, an improved understanding of this biological heterogeneity is being used to individualise adjuvant (postoperative) treatment. Traditionally, oncologists have relied on clinical and pathological tumour features as well as patient characteristics to determine risk of recurrence and to guide recommendations for chemotherapy. An evolving understanding of the molecular mechanisms that underlie the clinicopathologic features has led to the development of multiple molecular assays to help tailor therapeutic strategies for oestrogen receptor (ER) positive early breast cancer. In Ireland, since October 2011, the 21-gene Breast Recurrence Score® (Oncotype DX®) assay has been routinely used for patients with ER positive, lymph node negative early stage breast cancer. Prior work from our group suggested that access to this test in this population has led to a change in treatment recommendation in over half (59.2%) of all patients [2]. The change was primarily the omission of chemotherapy (in patients who would otherwise have received it), resulting in both reduced toxicity exposure and an estimated annual cost saving of approximately €500,000 [2].

Given the falling death rate from breast cancer in recent years (possibly in part due to a greater use of adjuvant chemotherapy), many physicians have been reluctant to abandon chemotherapy in the setting of involved lymph nodes. Since the number of involved lymph nodes correlates with a higher absolute risk of metastases, the absolute benefit of adjuvant chemotherapy in this setting is generally higher. In the RxPONDER trial, patients with ER positive breast cancer, involving up to 3 lymph nodes and Recurrence Score® results of ≤ 25, were randomised to chemotherapy with endocrine therapy or endocrine therapy alone [3]. This international study was open in Ireland and many patients were enrolled. After this study closed to accrual, there was no routine access to the Recurrence Score® testing. Many oncologists thought that broader access to this test would aid decision-making in clinics, prior to the results of the RxPONDER study being available. Therefore, this prospective, non-interventional study was conducted to determine and quantify the impact of the Recurrence Score® on the decision to recommend chemotherapy to patients with ER positive, lymph node positive early stage breast cancer in Ireland. The primary endpoint was the percentage reduction in the number of patients for whom chemotherapy was recommended following testing with the Recurrence Score®.

## Methods

This was a national, multi-site prospective, observational study conducted by Cancer Trials Ireland (CTRIAL-IE (ICORG) 15–34) in collaboration with Genomic Health Inc. (a subsidiary of Exact Sciences Corporation). Patients with oestrogen/progesterone receptor positive early breast cancer with one to three involved lymph nodes were eligible. The study was conducted across eight cancer centres and two additional hospitals and was approved by local ethics committees. Based on previous research, it was hypothesised that testing with the Recurrence Score® might reduce chemotherapy recommendations by up to 30%.

Following standard of care surgical resection and referral, patients attended a consultant medical oncologist and gave written informed consent for study participation. Patient characteristics including age, tumour characteristics and treatment details were recorded, including staging in accordance with the American Joint Committee on Cancer (AJCC) version eight. A questionnaire was completed by the consultant, detailing the estimated benefits of chemotherapy and the strength of recommendation for adjuvant cytotoxic chemotherapy. Testing with the Recurrence Score® was provided free of charge by Genomic Health, who also provided financial support for data collection. The Recurrence Score® result was classified as low (< 11), intermediate (11–25) and high (> 25) as defined in prospective clinical trials [7, 8]. Patients attended their consultant to discuss the test results and their consultant completed a second similar questionnaire, detailing the recommendations for adjuvant therapy given the Recurrence Score® result. Physician recommendations for chemotherapy pre-Recurrence Score® and recommendations post-Recurrence Score® were compared. Physicians were asked to make specific recommendations for chemotherapy regimens. The proportional reduction in anthracycline-taxane therapy, which are associated with the greatest toxicities, was quantified.

The sample size was based on similar decision impact studies conducted in other countries. The study was originally designed to include 75 patients who had undergone surgery and 75 tested in the preoperative setting. Due to slower than planned accrual, the preoperative cohort was closed early after the accrual of 12 patients.

## Results

Between March and September 2017, 75 patients were included across the ten hospitals. One patient had insufficient tumour tissue following surgery, and therefore, 74 patients were included in this study (Table 1). The median age was 54 (range 32–78) years and almost 70% of patients were 50 years of age or older. Ten patients (14%) had low risk Recurrence Score® results (< 11), 56 (76%) intermediate risk Recurrence Score® results (11–25) and eight (11%) high Recurrence Score® results (> 25). Overall, 40% had stage IIIA disease and approximately 70% and 10% in the intermediate and high risk Recurrence Score® results respectively. All six patients with grade 1 tumours had an intermediate risk Recurrence Score®. Almost 80% of patients with intermediate Recurrence Scores® had grade 2 tumours. There were six (40%) patients with a high risk Recurrence Score®, who had grade 3 tumours. The ratio of breast conserving surgery to mastectomy was almost 2:1, with similar proportions in each Recurrence Score® categories.

**Table 1:** Clinical characteristics by recurrence score result

Variable	Overall *N* (%)	Low test score < 11 *N* (%)	Intermediate test score 11–25 *N* (%)	High test score > 25 *N* (%)
Overall	74	10 (14)	56 (76)	8 (11)
Median age (range)	54 (32–78)	52	54	65
Age < 50	23 (31)	3 (13)	19 (83)	1 (4)
Age ≥ 50	51 (69)	7 (14)	37 (73)	7 (14)
Tumour grade				
Grade I	6 (8)	0 (0)	6 (100)	0 (0)
Grade II	53 (72)	9 (17)	42 (79)	2 (4)
Grade III	15 (20)	1 (7)	8 (53)	6 (40)
T-stage				
T1	32 (43)	4 (13)	23 (72)	5 (16)
T2	35 (47)	6 (17)	26 (74)	3 (9)
T3/4	7 (10)	0 (0)	7 (100)	0 (0)
Nodal status				
Nmi	14 (19)	1 (7)	13 (93)	0 (0)
1N	36 (49)	7 (19)	23 (64)	6 (17)
2N	13 (18)	2 (15)	10 (77)	1 (8)
3N	11 (15)	0 (0)	10 (91)	1 (9)
AJCC Staging – Eight Edition				
Stage I	2 (3)	0 (0)	2 (100)	0 (0)
Stage II	40 (54)	4 (10)	31 (78)	5 (13)
Stage III	32 (43)	6 (19)	23 (72)	3 (9)
Staging scans completed				
Yes	40 (54)	5 (13)	29 (73)	6 (15)
No	34 (46)	5 (15)	27 (79)	2 (6)
Breast surgery				
BCS	48 (65%)	6 (13)	36 (75)	6 (13)
Mastectomy	26 (35%)	4 (15)	20 (77)	2 (8)

Total percentages do not necessarily add to 100%, owing to percentage rounding

*mi* micrometastases, *BCS* breast conserving surgery

At study entry, oncologists recommend chemotherapy for 68 (92%) patients. Following testing for the Recurrence Score® result, chemotherapy was recommended for 48 (65%) patients, represented a 27% reduction in recommendation for chemotherapy. Of these, 44/48 (92%) patients had intermediate or high-risk results.

**Fig. 1:**
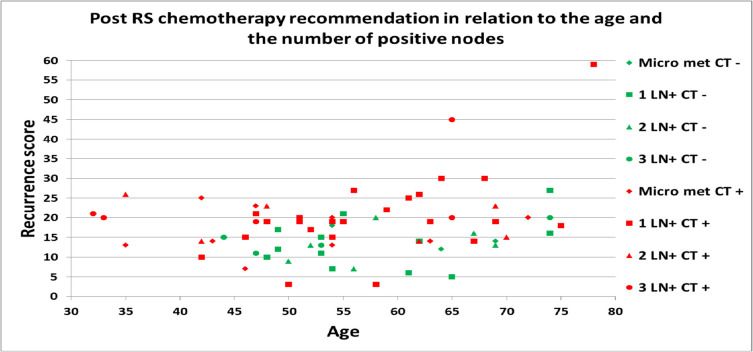
Recurrence Score result, age and lymph node (LN) count in patients recommenced chemotherapy (CT +) and non-chemotherapy regimens (CT-)

Chemotherapy was preferentially recommended for younger patients (Table 2): 37% of patients undergoing chemotherapy were < 50 years, and only 19% of those who did not opt for chemotherapy were aged < 50 years. Chemotherapy was recommended for all patients under the age of 40 (Fig. 1). There was one patient aged 78 with 1 involved lymph node (Recurrence Score® = 59) who was recommended chemotherapy and one aged 74 also with 1 lymph node (Recurrence Score® = 27), not recommended chemotherapy (Fig. 1).

**Table 2:** Clinical characteristics by cohort: pre- and post- recurrence score® testing

Variable	Overall *N* (%)	*Pre-* 21-gene test score chemotherapy recommendation *N* (%)		*Post-* 21-gene test score chemotherapy decision *N* (%)	
		Yes	No	Ye**s**	No
Overall	74	68 (92)	6 (8)	48 (65)	26 (35)
Age < 50	23 (31)	23 (100)	0 (0)	18 (78)	5 (22)
Age ≥ 50	51 (69)	45 (88)	6 (12)	30 (59)	21 (41)
Grade					
I	6 (8)	4 (67)	2 (33)	3 (50)	3 (50)
II	53 (72)	50 (94)	3 (6)	34 (64)	19 (36)
III	15 (20)	14 (93)	1 (7)	11 (73)	4 (27)
T-stage					
T1	32 (43)	31 (97)	1 (3)	25 (78)	7 (22)
T2	35 (47)	30 (86)	5 (14)	18 (51)	17 (49)
T3/4	7 (10)	7 (100)	0 (0)	5 (71)	2 (29)
Nodal status					
Nmi	14 (19)	12 (86)	2 (14)	11 (79)	3 (21)
1N	36 (49)	34 (94)	2 (6)	23 (64)	13 (36)
2N	13 (18)	13 (100)	0 (0)	7 (54)	6 (46)
3N	11 (15)	9 (82)	2 (18)	7 (64)	4 (36)
AJCC Staging – Eight Edition					
Stage I	5 (7)	4 (80)	1 (20)	5 (100)	0 (0)
Stage II	62 (84)	57 (92)	5 (8)	38 (61)	24 (39)
Stage III	7 (9)	7 (100)	0 (0)	5 (71)	2 (29)
Staging scans completed					
Yes	40 (54)	38 (95)	2 (5)	27 (68)	13 (32)
No	34 (46)	30 (88)	4 (12)	21 (62)	13 (38)
Breast surgery					
BCS	48 (65)	43 (90)	5 (10)	29 (60)	19 (40)
Mastectomy	26 (35)	25 (96)	1 (4)	19 (73)	7 (27)
21-gene recurrence score					
Low (< 11)	10 (14)	10 (100)	0 (0)	4 (40)	6 (60)
Intermediate (11–25)	56 (76)	51 (91)	5 (9)	37 (66)	19 (34)
High (> 25)	8 (11)	7 (88)	1 (13)	7 (88)	1 (13)

Total percentages do not necessarily add to 100%, owing to percentage rounding

*mi* micrometastases, *BCS* breast conserving surgery

**Fig. 2:**
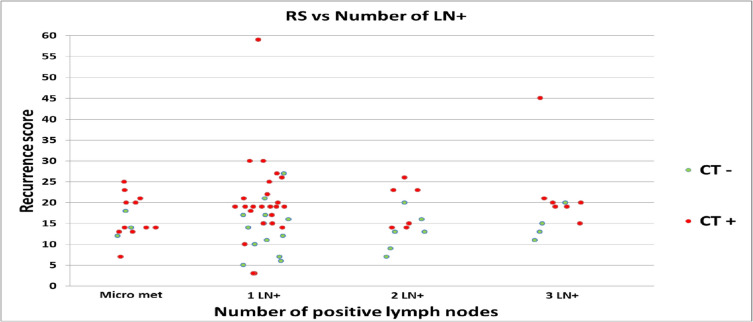
Recurrence Score (RS) result versus lymph node (LN) count in patients recommended chemotherapy (CT +) and non-chemotherapy regimens (CT-)

Most of the reduction in chemotherapy use was observed in patients with one involved lymph node (including micro metastasis) from 46 to 34 (16%) and in two lymph nodes from 13 to 7 (8%) (Fig. 2). The highest reduction in chemotherapy recommendations occurred in women between the ages of 51 to 70 with one lymph node involved, from 28 to 18 patients, representing a 36% reduction.

**Fig. 3:**
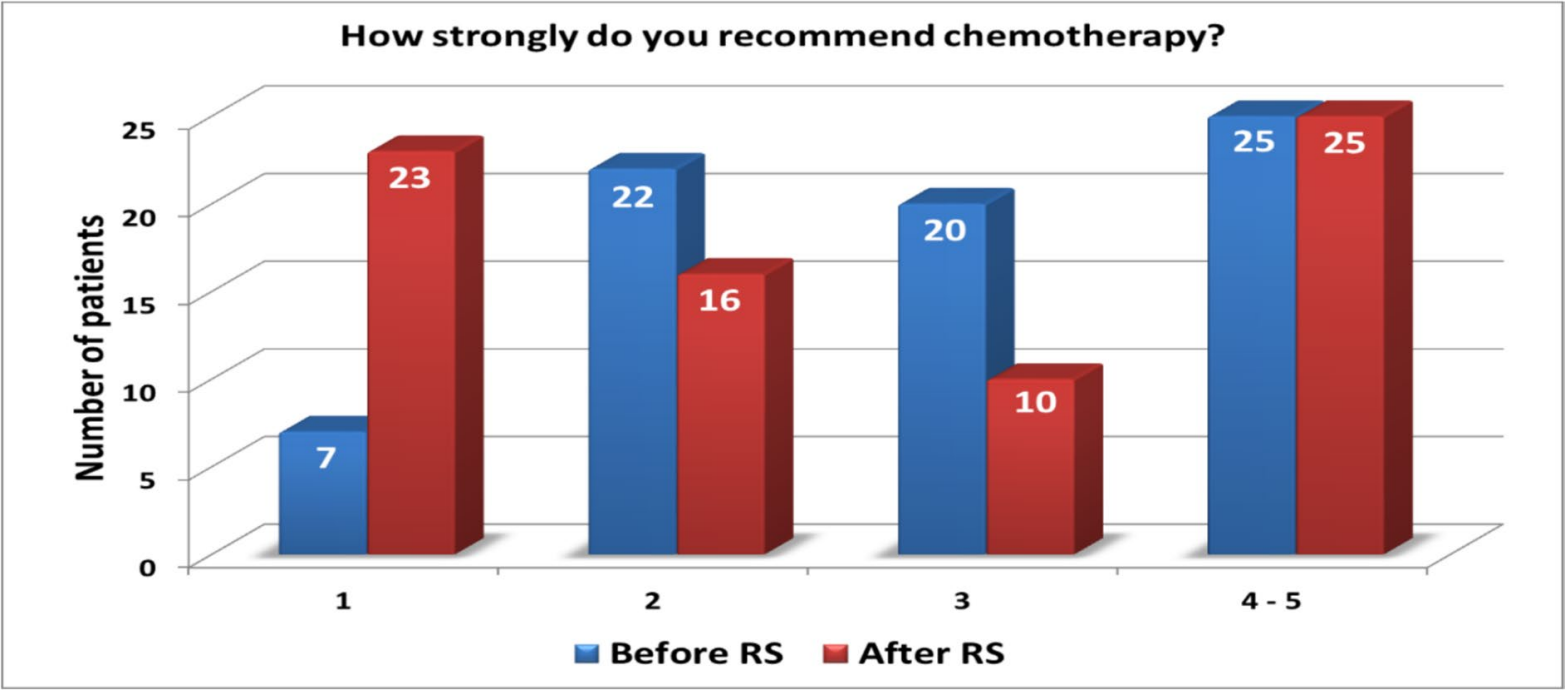
Opinion on strength of chemotherapy use before and after recurrence score (RS) result (1 = not very strongly/5 = very strongly)

**Fig. 4:**
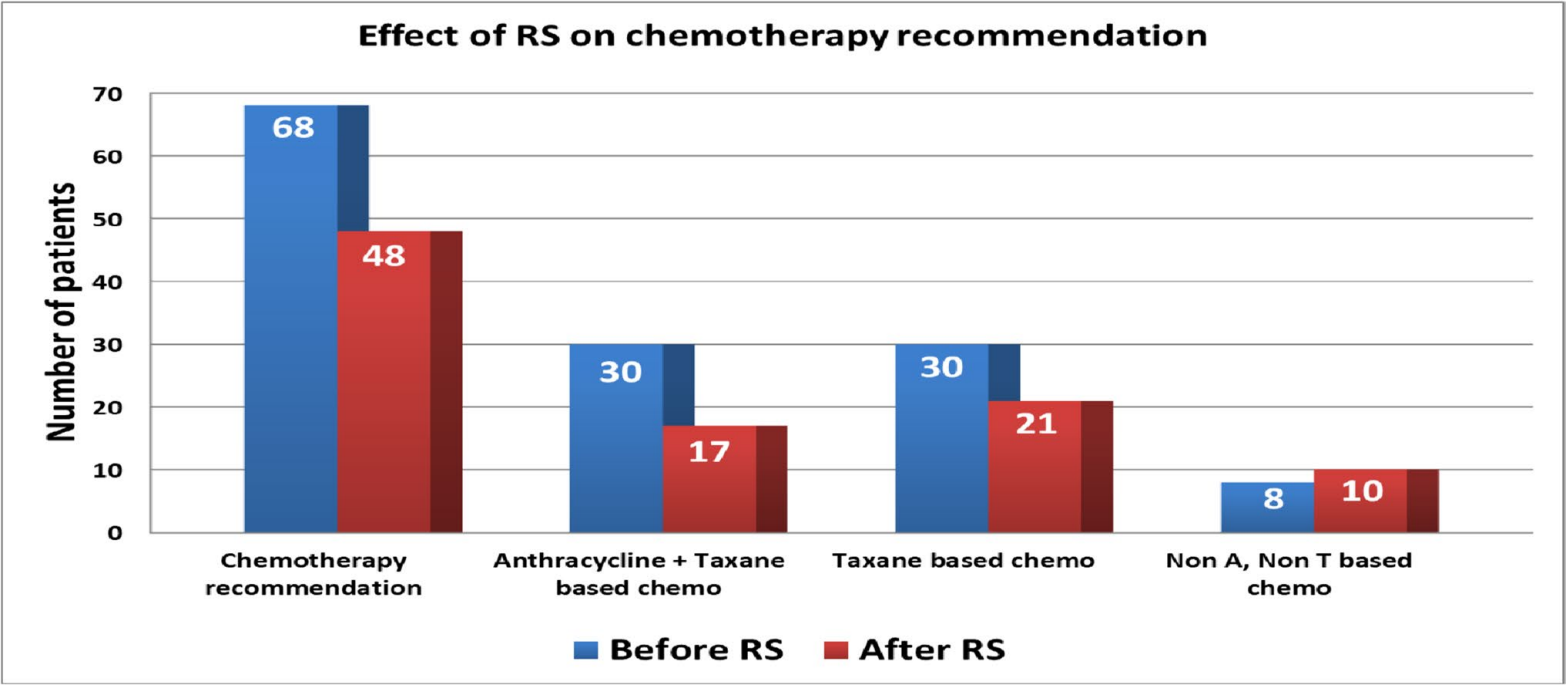
Change in number of patients recommended chemotherapy and regimens before and after recurrence score (RS) result. Note: A anthracycline; T taxane

The Recurrence Score® result also led to an increase in physicians’ perception of lack of tumour chemosensitivity with 57% and 64% reported they thought tumours were not very/not chemosensitive respectively. The proportion of patients for whom chemotherapy was strongly recommended (score of ≥ 3) reduced from 61 (*n* = 45) to 47% (*n* = 35) following Recurrence Score® testing (Fig. 3). Access to the Recurrence Score® test also led to physicians recommending less intensive chemotherapy (Fig. 4). Overall, there were 21 (31%) fewer patients recommended anthracycline-taxane combination or taxane-based regimens.

There were 23 patients aged under 50, of whom 18 had chemotherapy recommended based on their Recurrence Score® result (Table 3). Dose-dense AC-T (doxorubicin & cyclophosphamide with sequential paclitaxel) was the most preferred regimen in those with intermediate Recurrence Score® results (47%) followed by docetaxel & cyclophosphamide (TC, 26%). There were 51 patients aged 50 years and older, and taxane-based chemotherapy was the preferred regimen across each Recurrence Score® grouping (Table 4). There were 20 (27%) patients whose planned treatment changed as a result of Recurrence Score® testing (Table 5), including 6 with low risk results and 13 with intermediate risk scores.

**Table 3:** Chemotherapy recommendations by recurrence score result in patients aged < 50

Chemotherapy	Total *N* (%)	Low (< 11) *N* (%)	Intermediate (11–25) *N* (%)	High (> 25) *N* (%)
Anthracycline & Taxane^1^	10 (43)	0 (0)	9 (47)	1 (100)
Taxane-based^2^	7 (30)	2 (67)	5 (26)	0 (0)
CMF	1 (4)	0 (0)	1 (5)	0 (0)
No chemotherapy	5 (22)	1 (33)	4 (21)	0 (0)
Total	23 (100)	3 (100)	19 (100)	1 (100)

^1^ Includes doxorubicin and paclitaxel; ^2^ Includes docetaxel with cyclophosphamide and cyclophosphamide-methotrexate-5-fluorouracil (CMF) with paclitaxel regimens. Total percentages do not necessarily add to 100%, owing to percentage rounding

**Table 4:** Chemotherapy recommendations by recurrence score result in patients 50 years or older

Chemotherapy	Total *N* (%)	Low (< 11) *N* (%)	Intermediate (11 – 25) *N* (%)	High (> 25) *N* (%)
Anthracycline & Taxane^1^	14 (27)	2 (29)	10 (27)	2 (29)
Taxane-based^2^	23 (45)	4 (57)	15 (41)	4 (57)
CMF	8 (16)	1 (14)	7 (19)	0 (0)
No Chemotherapy	6 (12)	0 (0)	5 (14)	1 (14)
Total	51 (100)	7 (100)	37 (100)	7 (100)

^1^ Includes doxorubicin and paclitaxel; ^2^ Includes docetaxel with cyclophosphamide and cyclophosphamide-methotrexate-5-fluorouracil (CMF) with paclitaxel regimens. Total percentages do not necessarily add to 100%, owing to percentage rounding

**Table 5:** Change in treatment decision following 21-gene testing

Patients	*N*	Total change rate	Chemotherapy to no chemotherapy	No chemotherapy to chemotherapy
		*N* = 20	*N* = 19	*N* = 1
		*N* (%)	*N* (%)	*N* (%)
Test score				
Low (< 11)	10	6 (60)	6 (100)	0 (0)
Intermediate (11–25)	56	13 (23)	13 (100)	0 (0)
High (> 25)	8	1 (13)	0 (0)	1 (100)

## Discussion

This was the first prospective study evaluating the clinical impact of Recurrence Score® in women with hormone sensitive node positive early breast cancer in Ireland. Access to this test led to a decrease in the recommendation for chemotherapy in 27% patients, which was similar to the expected rate of 30%.

Other decision impact studies have demonstrated the clinical utility of early access to the Recurrence Score® results in patients with node positive disease. The current results are comparable with other similar sized prospective studies. In an Australian study of 101 patients with node negative and 50 patients with node positive disease, initial treatment recommendations were changed after receiving the Recurrence Score® result in 23% and 26% patients, respectively [5]. Similarly, in the SWITCH study [9], there was a 42% change in treatment, but this study was limited by a small number of patients with involved lymph nodes (*n* = 12). In the larger German study, of 122 patients with node positive disease, there was a 28% reduction in chemotherapy [10]. There was more grade 1 (55 versus 8%) and less grade 2 breast cancer (36 versus 72%) in the German study when compared with our study, but broadly these results are extremely consistent. One limitation of these previously published studies was the inclusion of patients with node negative disease. A survey of 160 medical oncologists in the USA revealed that 51% of patients with ER positive, node positive breast cancer had their treatment recommendation changed after receipt of a Recurrence Score® result [6]. In all of these and other studies, the use of the Recurrence Score® led to an overall decrease in recommendations for chemotherapy [4–6].

There were a number of other important findings in our study. As might have been expected, the reduction in chemotherapy recommendation was greatest in those with lower risk Recurrence Score® results. Access to the test also led to the use of less intensive chemotherapy—there were 13 fewer patients (18%) prescribed anthracycline-taxane combination chemotherapy and nine fewer patients (12%) requiring taxane-based chemotherapy regimens.

One of the strengths of this study includes is that it is representative of patients across the eight national cancer centres. The population study size is similar to other international studies conducted in the same population. However, this study was not conducted to assess the validity of the Recurrence Score® assay, In fact, since this study was conducted, the RxPONDER study has prospectively validated the use of the Recurrence Score® assay in patients with 1–3 positive lymph nodes [3]. As a result of this study and others, many older postmenopausal women are now spared chemotherapy. Conversely, the RxPONDER study did not identify any group of younger patients who could avoid chemotherapy and novel therapies are needed for these women.

In conclusion, this study demonstrates that early access to the Recurrence Score® assay influenced the treatment plan of Medical Oncologists in Ireland. This resulted in the omission of chemotherapy for certain patients and in many cases the use of less toxic chemotherapy. Early access to cancer genomic tests can facilitate more individualised treatment for patients.
